# Predicting Intraocular Pressure From Glaucoma Patients Receiving Medication Treatment Using Explainable Machine Learning

**DOI:** 10.1155/bmri/9930837

**Published:** 2026-01-30

**Authors:** Robert T. James, Wenke Liu, Gadi Wollstein, Joel S. Schuman, David Fenyo, Kevin C. Chan

**Affiliations:** ^1^ Departments of Ophthalmology and Radiology, Tech4Health Institute and Neuroscience Institute, New York University Grossman School of Medicine, NYU Langone Health, New York University, New York, New York, USA, nyu.edu; ^2^ Institute for Systems Genetics, New York University Grossman School of Medicine, NYU Langone Health, New York University, New York, New York, USA, nyu.edu; ^3^ Wills Eye Hospital, Philadelphia, Pennsylvania, USA, willseye.org; ^4^ Drexel University School of Biomedical Engineering, Science and Health Systems, Philadelphia, Pennsylvania, USA; ^5^ Department of Biomedical Engineering, Tandon School of Engineering, New York University, Brooklyn, New York, USA, nyu.edu; ^6^ Spencer Center for Vision Research, Byers Eye Institute, Department of Ophthalmology, Stanford University School of Medicine, Palo Alto, California, USA, stanford.edu

**Keywords:** explainable machine learning, glaucoma, insulin-like growth factor 1 (IGF-1), intraocular pressure (IOP), low-density lipoprotein (LDL)

## Abstract

Glaucoma is a chronic neurodegenerative disease of the visual system, and treatment is targeted toward lowering intraocular pressure. However, some patients fail to respond to treatment and their intraocular pressure levels remain high, risking continuous vision loss. Explainable machine learning provides a mechanism for both individual prognostication and the identification of factors associated with treatment outcome. Here, we used explainable machine learning to predict intraocular pressure for glaucoma patients receiving medication treatment. We accessed the UK Biobank to obtain information on 290 eyes from 161 participants who reported a diagnosis of glaucoma and were receiving treatment. Features were divided into three distinct datasets containing demographic data only, physiometabolic parameters and medication prescription data, and all data combined. We evaluated five machine learning techniques for each feature set in terms of their ability to predict intraocular pressure at a follow‐up visit in a classification task. We then calculated SHapley Additive exPlanation (SHAP) values for the best performing model to determine feature importance, stability, and interactions. We found that eXtreme Gradient Boosting (XGBoost) outperformed all other models when trained and tested on the combined feature set with an area under receiver operating characteristic curve (AUC) of 0.708. Insulin‐like growth factor 1 (IGF‐1), low‐density lipoprotein (LDL), and lymphocyte count ranked as the three most important features for this model. LDL and IGF‐1 exhibited a low degree of global variability in contribution to the model output across all cross‐validation repeats. SHAP values demonstrated the strongest interactions being between LDL and IGF‐1. In summary, our studies indicated the importance of blood LDL and IGF‐1 in contributing to the outcomes of intraocular pressure lowering treatment and demonstrated the ability of XGBoost to predict these outcomes.

## 1. Introduction

Glaucoma is a chronic, progressive optic neuropathy and the foremost leading cause of irreversible blindness globally, affecting approximately 76 million people in 2020 [1]. Management of glaucoma is targeted towards lowering intraocular pressure (IOP) but there remains a subset of patients who do not achieve an optimal IOP[2]. To date, few studies have employed machine learning and artificial intelligence to predict glaucoma treatment outcomes and identify the features most associated with accurate prognosis [3–5]. One such study found that a decision tree model obtained an area under the receiver operating characteristic curve (AUC) of 0.710 when predicting whether patients would have elevated IOP within 1 month of receiving surgery for glaucoma after training on variables related to demographics, medication prescription, and eye exam data [6]. A similar recent study attempted to predict trabeculectomy outcomes based on systemic health, ocular, and demographic data and found a random forest model to perform with an AUC of 0.680 [7]. Another study collected demographic and systemic health data on 385 patients with glaucoma and found a multivariable logistic regression to be the best performing model with an AUC of 0.610 when determining whether patients would need surgery [8]. These emerging studies, although promising, imposed several limitations. For example, some studies used endpoints other than IOP for prognostication. Furthermore, several of these studies used mean decrease in accuracy and mean decrease in impurity to interpret their models. Both methods assume that all features are independent, and thus cannot capture feature interactions [9]. In this study, we conducted a similar investigation as those mentioned previously on a cohort of 290 patients from the UK Biobank, utilizing a wider range of features obtained from blood tests, and assessed their interactions. Features were divided into three distinct sets and we applied five machine learning algorithms to each feature set to compare their performance in predicting the presence of elevated versus normal IOP (> or < 21 mmHg, respectively) at a follow‐up visit.

## 2. Materials and Methods

This research has been conducted using the UK Biobank Resource under Application Number 91781. The UK Biobank is an ongoing prospective longitudinal study holding a wide array of data on over 500,000 participants aged 40–69 years. All participants were recruited between 2006 and 2010 while living within 25 miles of at least one of 22 assessment centers positioned at various locations across the UK. At their initial visit, information was collected through self‐reporting questionnaires, verbal interviews, physical measurements, and biological specimens. Data collected through questionnaires and verbal interviews include sociodemographic factors along with past medical and social history. Biological specimens were used to conduct lab tests on blood, saliva, and urine samples. Participants were then contacted for a follow‐up visit between 2012 and 2013 for repeat measurements of all previous measures.

We accessed the UK Biobank to analyze a subset of patients who self‐reported a diagnosis of glaucoma at their initial visit. All data was extracted from the UK Biobank based on a snapshot of availability in April 2023. We formulated a comprehensive list of glaucoma treatment medications that were available at the time of the study′s data collection by both brand name and trade name, which fall into one of five treatment classes: prostaglandin analogs, beta blockers, carbonic anhydrase inhibitors, alpha‐adrenergic agonists, and cholinergic agents. We further narrowed the cohort to only include patients who reported taking at least one medication in the list. We also determined which eye of each patient was diagnosed with glaucoma at both baseline and follow‐up visits. If a patient reported having glaucoma at baseline but data was not available pertaining to eye laterality until the follow‐up visit, it was assumed the laterality reported at follow‐up was also indicative of the laterality at baseline. We excluded patients if there was no information pertaining to the eye laterality at both baseline and follow‐up. We also excluded patients who did not have an IOP measurement at their follow‐up visit. Only one IOP measurement was available for all participants at the follow‐up visit.

We selected variables from the UK Biobank to be used as features based on availability within the databank as well as previously studied factors potentially linked to glaucoma development or progression. We arbitrarily grouped these features into three datasets: (1) demographic and baseline health (DBH), (2) physiometabolic + medication prescription, and (3) a combined feature set containing all variables. The DBH feature set was composed of 14 features encapsulating patients′ age, sex, past medical history, and past social history. The physiometabolic and prescription feature set encompassed 28 features relating to physical measures such as blood pressure and pulse rate along with various blood‐based lab tests and variables describing the class of glaucoma treatment to which a patient′s medication belonged. We created dummy variables for all categorical features with more than two values within the feature. Features containing continuous variables were normalized with zero mean and unit standard deviation. All features were gathered at each patient′s initial visit.

We selected five machine learning models to perform a binary classification task to predict whether a patient would have elevated or normal IOP measured at their follow‐up visit. We classified any corneal‐compensated IOP greater than 21 mmHg as being elevated, whereas measurements below this value were categorized as normal. We used corneal‐compensated IOP instead of Goldman‐correlated IOP as there is evidence that corneal compensated measurements are more resistant to factors such as corneal resistance, thickness, and hysteresis that may confound IOP values [10]. The five models evaluated in this study include two gradient boosted machine learning algorithms, a random forest decision tree model, a support vector machine, and a linear or logistic regression model for the regression and classification tasks, respectively. We focused on tree‐based methods due to their interpretability, flexibility for input features and wide applications in biomedical research. The packages we used for the gradient boosted models were eXtreme Gradient Boosting (XGBoost Version 2.0.3) and Light Gradient Boosting Machine (Light GBM Version 4.2.0). We used the scikit‐learn Version 1.4.0 random forest classifier to complete the classification task. In order to compare performance of these models to relatively basic models, we also constructed a logistic regression model for the classification task from the scikit‐learn (sklearn Version 1.4.0) library.

To identify which machine learning algorithm has the best performance in the classification task, we utilized a nested cross‐validation procedure. This procedure relays the benefit of determining the most optimal hyperparameters for each model as well as giving an estimate of generalization error when not enough data is available for a traditional train/test split where a portion of data is reserved for testing model performance while the rest is used for the purpose of training. Such a split would, in cases where an algorithm must be trained on a small amount of data, lead to an overly optimistic estimation of performance. Thus, the cross‐validation procedure gives a more realistic estimate of true model performance if it were trained and tested on external data. A basic form of cross‐validation known as *k*‐fold cross‐validation partitions the entire set of data into *k* separate sets, or folds, of almost equal size. A model is then trained on *k* − 1‐folds and validated on the remaining *k* ^th^ fold. This process is repeated *k* times, with each fold serving as the validation set once and every observation appearing at least once in both the training and testing fold. The performance metrics are then averaged across all iterations to provide a more reliable estimate of the model′s generalization ability. Nested cross‐validation entails the use of an outer loop and an inner loop. The outer cross‐validation loop estimates the performance of the inner cross‐validation method of hyperparameter tuning and model selection. This diminishes the bias inherent in nonnested cross‐validation as the test data in the outer folds are not used to train the hyperparameter‐tuned models that were fitted in the inner fold. To conduct a comprehensive search through parameter space and identify the best hyperparameters for each model across each feature set, we employed the RandomizedSearchCV package from the scikit‐learn library, a widely used machine learning framework. This package performs a randomized search over the specified hyperparameter space and samples combinations of hyperparameters to evaluate the model’s performance using a threefold cross‐validation strategy. After selecting the best models based on inner loop performance, we evaluated the performance of each model on the entire dataset via the outer‐loop threefold cross‐validation. We averaged the AUC scores and summarized them in a bar chart.

SHapley Additive exPlanation (SHAP) value is a model‐agnostic metric that evaluates the contribution of each feature to the prediction of a single data point. The SHAP interaction value further measures additional contributions when a pair of features are considered together. We used the SHAP python package Version 0.44.1 to calculate SHAP values for the best performing classification model. To provide a more robust estimate of the contribution of features to model performance, we performed a 10x repeated threefold cross‐validation where SHAP values were extracted for each sample per cross‐validation and averaged across all repeats into one value for plotting and visualization. However, averaging the SHA*P* values in this manner may obscure variability, making it difficult to determine the degree to which SHAP values for a given feature varied across different cross‐validation data splits. To account for this, we used two separate methods to calculate the stability of feature importance. One method includes the use of the Shapash package in python to calculate the local variability in SHAP values for closely related neighboring instances. The *N* nearest neighbors of a given instance are selected for comparison if they are within an L1 distance of 10 and their predicted output is within 10%. After the neighbors are selected, a stability metric is calculated for all neighbors by taking the ratio of the standard deviation of the normalized vectors of the feature contributions to the average of the absolute values of the normalized vectors of the feature contributions. We evaluated the stability metric for all neighborhoods that met the criteria. We also constructed a global variability scatter plot to visualize the range of SHAP values for each feature across all participants (or sample) per cross‐validation repeat. Essentially, this is plotting the maximum averaged SHAP value for a given feature value for a participant minus the minimum SHAP value observed during any of the 10 repeated cross‐validations. This assists in gauging the stability of feature importance across all repeats.

## 3. Results

We gathered information on 290 eyes from 161 participants. Ninety‐eight eyes (33.8%) were classified as having elevated IOP at follow‐up, whereas 192 eyes (66.2%) were classified as having normal IOP. One hundred and twenty‐six eyes (43.4%) were from female participants and 164 (56.5%) were from male participants. We also found that male participants were significantly more likely to have an eye with elevated IOP than female participants (*p* = 0.012).

The final hyperparameters and their respective values for each dataset a given model was trained and tested on are depicted in Tables S1, S2, S3 and S4. XGBoost outperformed all other models when trained and tested on the combined feature set containing all features with an AUC of 0.708 (Figure 1a). This finding demonstrates that there are statistical patterns learned by this model when analyzing data from all features that are not present when they are split. The top feature alone could explain 90% of the model output for 77.1% of all samples in the dataset (Figure 1b). Only the Top 4 features were needed to explain 90% of the model output for all (100.0%) samples of the dataset. Thus, a 90% accurate explanation about the model can be given with a small subset of features for the vast majority of samples in the dataset. However, the Top 4 features are not sufficient to provide a 100% accurate explanation of model output for more than 10% of the samples in the dataset (Figure 1c). This is likely because XGBoost learned patterns that required the presence of features which on average had less contributions to the model output but were still useful for a minority of instances in the dataset.

Figure 1Classification model performance and compacity of explanations for XGBoost, the best performing classification model when trained on the combined feature set. (a) Bar chart of average threefold cross‐validation receiver operating‐characteristic area under curve (AUC) score for each machine learning algorithm for the classification task across feature sets. Standard error bars are included. LGBM, light gradient boosting machine; XGB, XGBoost; SVM, support vector machine classifier; RF, random forest tree classifier; LR, logistic regression. (b) The minimum number of required features to approximate 90% of our XGBoost model output starting with the most important feature and adding other features stepwise over a cumulative proportion of the entire dataset. This data is only in reference to the XGBoost model alone as that was the best performing model in the classification task. For this model, the top feature was needed to explain 90% of the model output for 77.1% of all samples in the dataset. The Top 4 features were needed to explain 90% of the model output for all (100.0%) samples of the dataset. (c) The level of our XGBoost model approximation achieved from use of only the Top 4 features across a cumulative distribution of samples. This data is only in reference to the XGBoost model alone as well. The Top 4 features provide 90% of model approximation for 100% of samples in the dataset and provide 100% of model approximation for 10.8% of samples in the dataset. For an ordered list of the most important features, please see Figure 2a.

**Figure:**
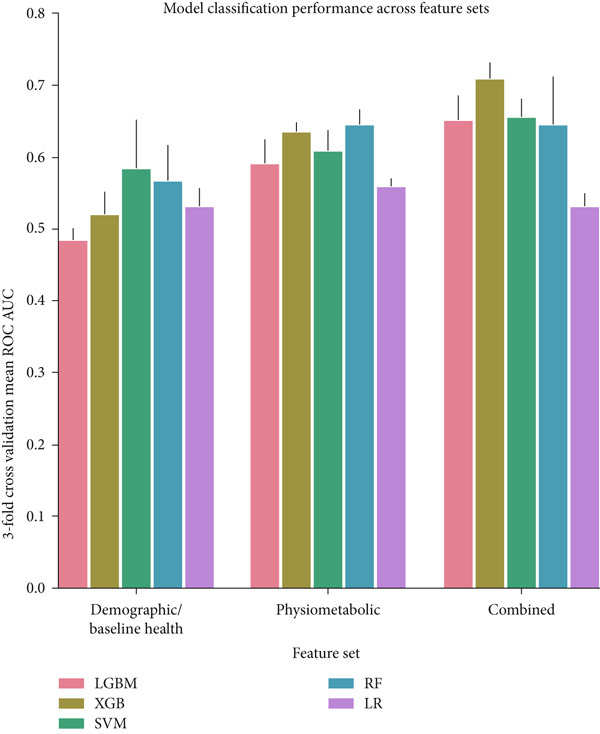
(a)

**Figure:**
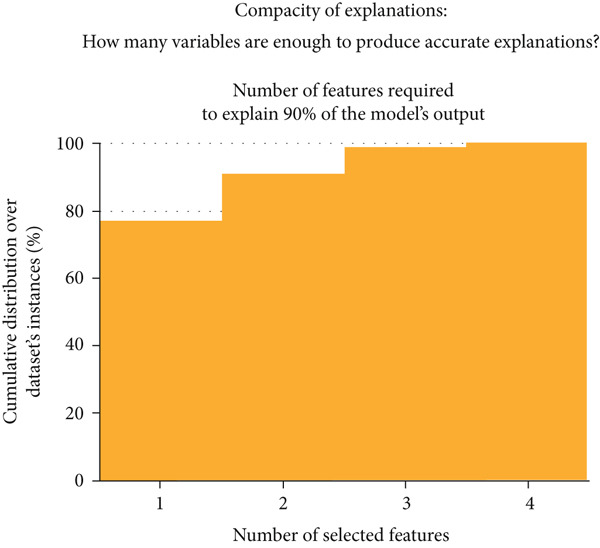
(b)

**Figure:**
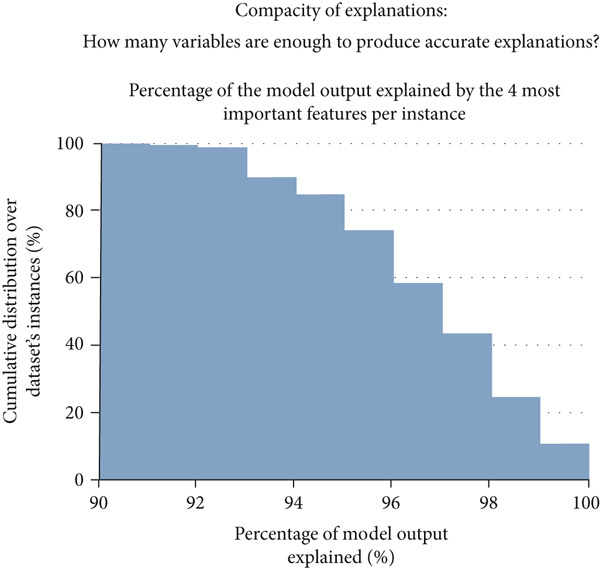
(c)

Insulin‐like growth factor 1 (IGF‐1), low‐density lipoprotein (LDL), and lymphocyte count rank as the three most important features for XGBoost when trained on the combined feature set (Figure 2a). Features that have less importance beyond the Top 5 features tended to have SHAP values that were clustered near the null effects line (i.e., SHAP value of zero), meaning a large portion of the values for these features had little to no effect on model output. All features with the exception of one in the Top 15 were physiometabolic features, suggesting that these features provided the most contributions to model output and were more important than the demographic or baseline health features. Both high and low feature values had similar SHAP values in both a positive and negative direction alike. Thus, differing feature values have similar effects on the model when there is a prediction of a positive case (elevated IOP) and a negative case (normal IOP).

Figure 2(a) The distribution of averaged SHA*P* values across all 10 repeats of the threefold outer loop cross‐validation for XGBoost, the best performing classification model when trained and tested on the combined feature set. Features are ranked in importance from top to bottom. (b) A SHAP global variability plot showing the mean scaled range (maximum–minimum) of SHAP values for the Top 14 features per participant across all 10x repeated threefold cross‐validations for XGBoost trained and tested on the combined dataset. (c) A SHAP local variability plot using the *N* nearest neighbor method. An arbitrary cutoff for normalized local variability of 0.1 was used to denote the upper bounds of a stable feature and 0.3 to delimit the upper bounds of unstable features. Each data point represents the normalized local contribution value variability, or the stability metric, for a given neighborhood of samples that satisfy the criteria of similarity. Features are listed from top to bottom in terms of their importance as calculated by the sum of SHAP values.

**Figure:**
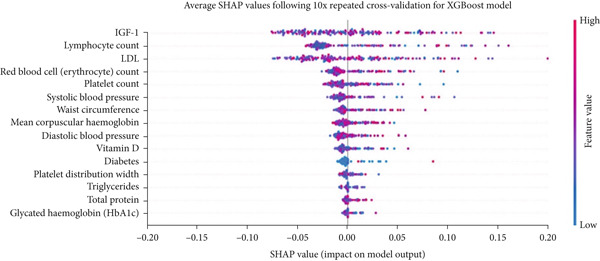
(a)

**Figure:**
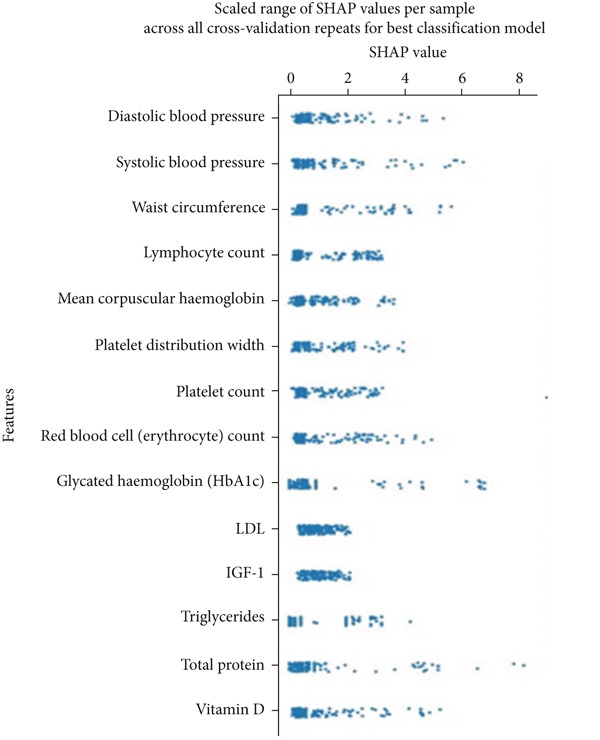
(b)

**Figure:**
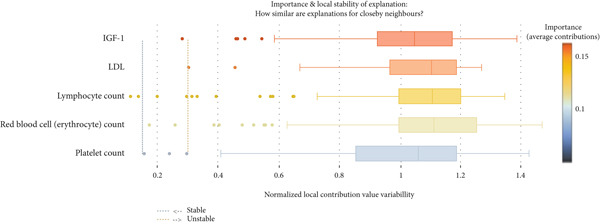
(c)

The global variability scatterplot (Figure 2b) demonstrated that in relation to all other features, only LDL and IGF‐1 exhibited a relatively low degree of variability in contribution to the model output across all cross‐validation repeats. This suggests that they have relatively stable and robust feature importance for our XGBoost model across the entire dataset. The local variability boxplot (Figure 2c) revealed substantial variability in feature importance within samples of a neighborhood. The most important features had a stability metric above an arbitrary threshold of 0.10, which is the upper bound of stable feature importance, suggesting that these features are regionally unstable and vary based on the instance the model is making a prediction for.

**Figure 3:**
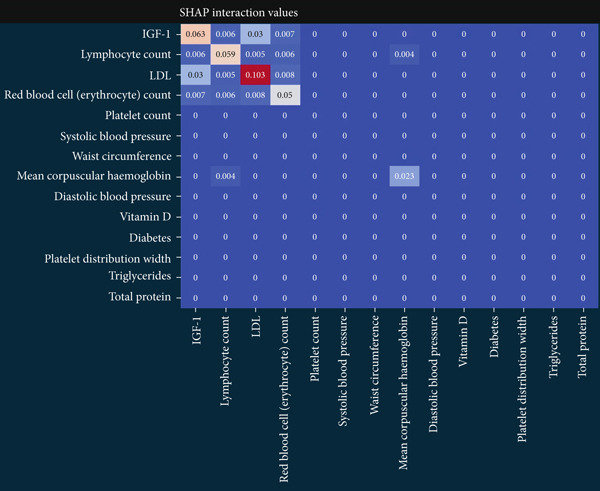
A matrix of interaction and main effect values calculated via SHAP for the Top 14 features in the combined feature set. The diagonal features are main effect values which capture the effect that the value of a given feature alone has on model output after subtracting the additional feature interactions. The off‐diagonal values represent the additional effect on model output that occurs between pairwise interactions between features.

The matrix of calculated SHAP interaction values demonstrates few interactions between features, with the strongest being between LDL and IGF‐1 (Figure 3). The dependence plots for IGF‐1 and LDL (Figure 4a,b) show the lack of a clear correlation between the magnitude of the values of these features and the effects they have on the model output. However, these dependence plots reveal considerable interaction effects between the two features and other features in the dataset as demonstrated by the vertical spread of data points. This spread represents how the value of a feature alone is not driving model output, but rather the main effect of the feature value along with the added effect of feature interactions contribute to model output. Further analysis reveals that the main effect (after subtracting the interaction effects) of both IGF‐1 and LDL values is correlated with their respective feature values (Figure 4c,d). IGF‐1 values below 25.0 nmol/L contributed to a classification of normal IOP, whereas IGF‐1 values above this threshold contributed to a classification of elevated IOP for our XGBoost model. LDL had similar thresholds where the main effect on model output switches but at multiple boundaries. When evaluating the pairwise interaction values for both LDL and IGF‐1, there exist some correlations between the values of these features and the effect on model output. Below the 25.0 nmol/L value for IGF‐1, if the LDL was above 3.5 mmol/L then these features interacted to contribute to a prediction of elevated IOP, whereas an LDL less than 3.5 mmol/L contributed to a prediction of normal IOP (Figure 4e). Above the 25.0 nmol/L value for IGF‐1, lower LDL levels were correlated with a prediction of elevated IOP, and higher levels of LDL were correlated with a prediction of normal IOP. A matrix of interaction and main effect values calculated via SHAP for all features in the combined feature set can be found in Figure S1.

Figure 4(a, b) Scatterplots representing the relationship between IGF‐1 and LDL feature values and their corresponding Shapley contribution values as calculated by SHAP. Vertical dispersions of data show that the same feature value has different SHAP contributions due to the presence of feature interactions. (c, d) The main effect that IGF‐1 and LDL values have on SHAP contribution values after subtracting feature interaction values from the overall SHAP contributions for each sample. (e) The distribution of feature interaction values across both IGF‐1 and LDL values.

**Figure:**
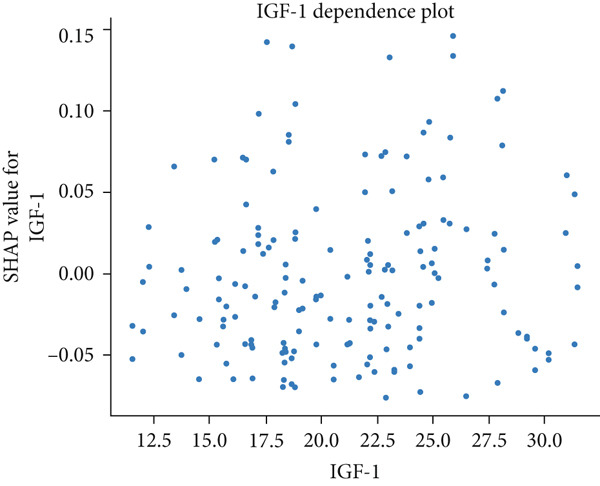
(a)

**Figure:**
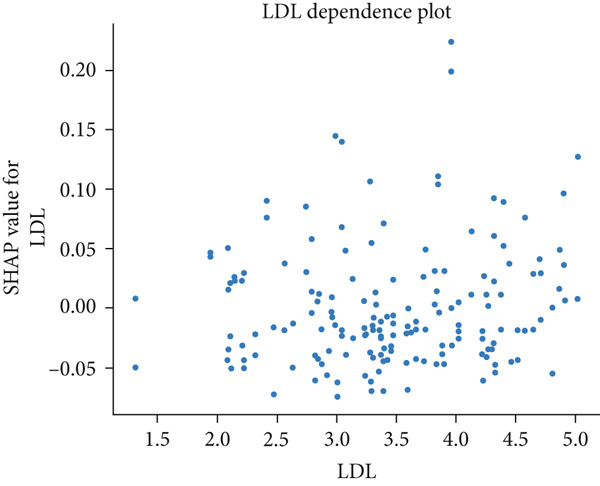
(b)

**Figure:**
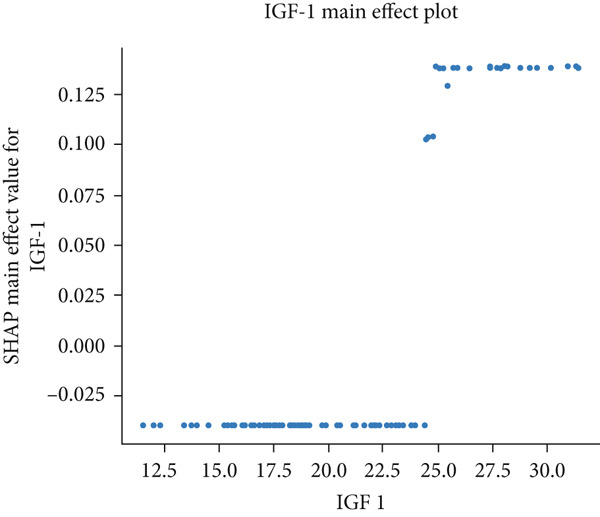
(c)

**Figure:**
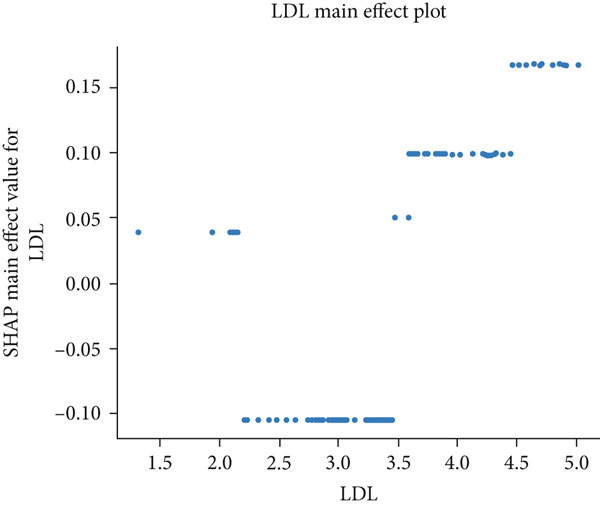
(d)

**Figure:**
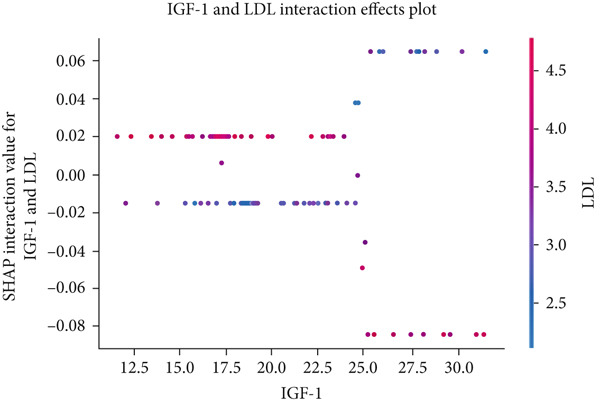
(e)

## 4. Discussion

We evaluated the performance of five machine learning techniques in the classification task of predicting IOP status in a subset of glaucoma patients who reported taking at least one of five classes of therapeutics. It is noteworthy that XGBoost outperformed Light GBM, another gradient‐boosted decision tree package. Unlike XGBoost, Light GBM uses a leaf‐wise tree growth strategy, allowing the decision tree to reach a high degree of complexity which can lead to overfitting. This complication is further exacerbated when there is limited data on which to train Light GBM, as is the case in this study. XGBoost also demonstrated a lower standard deviation in classification performance than Light GBM, potentially due to its resistance to overfitting. All models achieved greater performance than the logistic regression model, likely because the classification task in this study is complex while feature interactions, which basic logistic regression models cannot take into account without explicit implementation, are important for learning the relationship between the features in this study and IOP.

After identifying the best performing model, we summarized feature importance with the SHAP package. We then examined the stability of such feature importance in two ways. One way is to find the difference between the maximum and minimum SHAP values of a given feature for each participant across all repeat cross‐validations and visualize this range in a scatter plot. The other way utilizes a nearest neighbor method where samples that are within a given distance from each other in feature space and have received similar outputs from XGBoost are examined for their variation in SHAP values for a given feature. While we did not list the top features for each cross‐validation fold or each repeat of cross‐validation, it can be surmised from these two methods that the most important features overall varied across each cross validation. For example, the average stability for the most important features using the nearest neighbors method was above an arbitrary threshold of 0.10 which was deemed to be the upper bound of “stable” feature importance. This means that feature importance varied quite broadly based on the instance our XGBoost model is making a prediction for. The scatterplot qualitatively depicts the variation in feature contributions in a more global sense for our XGBoost model, considering all instances, as opposed to only closely related instance. This method illustrates LDL and IGF‐1 as being relatively more stable features than all the others (i.e., less global variability in SHAP values across repeat cross‐validations). However, as noted with the nearest neighbors method, focusing on a smaller subset of the data reveals greater local variability of importance for these features.

There exists within the literature some evidence that IGF‐1 plays a role in glaucoma pathogenesis. Proteomic analysis of aqueous humor showed lower IGF‐1 levels in glaucoma patients compared to cataract patients without glaucoma [11]. Animal studies also support the role that IGF‐1 plays in retinal ganglion cell survival and antiapoptotic processes [12]. However, the correlation between glaucoma and IGF‐1 is not always unidirectional. Investigations have revealed higher rates of increased IOP and glaucoma in patients with acromegaly, a condition characterized by excess endogenous growth hormone production and high IGF‐1 levels. Children who are deficient in growth hormone production and resultantly treated with recombinant human growth hormone therapy were also found to have increased IOP in a dose‐dependent manner [13]. After intravenous administration of arginine, glaucoma patients without acromegaly showed higher levels of plasma human growth hormone than healthy controls [14], whereas arginine can stimulate the production and secretion of IGF‐1 both directly and indirectly through growth hormone [15]. In our study, we found no difference in IGF‐1 serum levels between patients with normal and elevated IOP at follow‐up (normal = 21.73 ± 5.30 nmol/L; elevated = 21.90 ± 5.30 nmol/L; *p* = 0.145). A study comparing serum IGF‐1 levels in patients with pseudoexfoliative glaucoma versus controls also found no significant difference between groups [16]. Our study did not evaluate the mechanism and subtype of glaucoma that patients were diagnosed with. Further studies are needed to determine if different subtypes of glaucoma have IOP levels that are more associated with serum IGF‐1 values than others with varying feature importance.

Regarding the association between LDL and glaucoma, LDL was found to positively correlate with mild increases in IOP, while patients taking LDL‐lowering medications may imply a lower risk of glaucoma [17–19]. It is hypothesized that hyperlipidemia can increase blood viscosity leading to an increased episcleral venous pressure and a subsequent rise in IOP [20]. Increased circulating LDL can also contribute to retinal vasculature atherosclerosis, and the resultant narrowing of retinal vessels can raise the risk of glaucoma. On the contrary, there is research on the UK Biobank demonstrating a higher risk of glaucoma with lower LDL levels [21]. In our subset of patients from the UK Biobank, patients with elevated IOP had similar blood LDL levels as patients with normal IOP at follow‐up (serum LDL level with normal IOP = 3.50 ± 0.90 mmol/L; serum LDL level with elevated IOP = 3.70 ± 0.90 mmol/L; *p* = 0.157). Another study also found no significant difference in the LDL level between normal tension glaucoma patients and healthy controls [22]. These variable correlations between LDL, IOP, and glaucoma reflect the mixed and complex relationships between LDL and glaucoma, as well as the need for further research to elucidate the effect that LDL has on the development and progression of glaucoma.

In our study, the lack of an apparent correlation between IOP and IGF‐1 or LDL, the most stable and important features, suggests that our XGBoost model is taking into account the interactions between features to make its predictions. Feature interaction analysis showed that the greatest feature interactions occurred between LDL and IGF‐1. It was also demonstrated that a stepwise change in both the main effects and interaction effects of these features existed, suggesting that XGBoost learned decision boundaries within and between these two features. It cannot be demonstrated that these two features have a causal relationship with IOP. Nonetheless, our XGBoost model learned a nonlinear statistical relationship between LDL, IGF‐1, and IOP in a manner that allowed it to perform with an AUC of 0.709, suggesting a possible link between these variables and IOP in glaucoma patients that has not been fully elucidated by current research.

The performance of our XGBoost model was similar to other models that attempted to predict IOP or a closely related proxy [4, 6, 7]. Lin et al. found that their XGBoost model, when trained on similar metabolic features, achieved an AUC score of 0.710 in predicting whether a patient would have elevated IOP 1 month posttrabeculectomy procedure [6]. Similarly, Banna et al. found that their random forest classifier model obtained an AUC score of 0.680 when trained on demographic, systemic health, and ocular data in the task of predicting whether patients would have success or failure 1 year posttrabeculectomy [7]. In comparison to our study, the models engineered by Lin et al. and Banna et al. did not train on as wide a range of metabolic data gathered from blood‐based lab tests, nor did these studies attempt to explain the behavior of their models with SHAP. Notably, a recent XGBoost model by Barry and Wang′s was able to achieve an AUC score of 0.855 when tasked with predicting whether a glaucoma surgery would be a failure based on inability to decrease pre‐operative IOP by 80% [4]. However, its performance decreased to an AUC score of 0.665 when the model was tasked with predicting surgical failure when the surgical failure threshold was changed to a postoperative IOP reduction of 20% or a postoperative IOP < 21 mmHg. Unlike Barry and Wang′s model, our XGBoost model did not include prior measurements of IOP as a feature for training, thus making our model more suitable for predicting outcomes when prior IOP measurements are sparse or unavailable. Although it remains inconclusive whether the current level of model accuracy is sufficient for potential clinical applications, this work represents a proof‐of‐concept illustration of the predictive capabilities of our model and how this lays the groundwork for determining the efficacy of IOP‐lowering therapy through topical ophthalmic solutions for individual patients. Future studies may test if machine learning can aid clinicians in deciding between IOP‐lowering medical therapy versus surgery or minimally invasive laser procedures such as selective laser trabeculoplasty in cases where prognosis is not clear but a patient may be at a high risk of progression.

There are several limitations to the findings of this study. This study included a small sample of eyes and lacked an external dataset for validation. It is possible that if given the same set of parameters on a larger cohort with samples ordering in the thousands or on a different sample of participants of the same size, model performance and feature importance could be altered. However, the nested cross‐validation procedure was used to provide the best estimate of generalization error and to approximate the model′s performance if trained and tested on larger sample sizes or datasets of commensurate size but externally. In keeping with recent methods of research involving machine learning, the purpose of the nested cross‐validation procedure is to simulate the use of external validation datasets especially when the availability of external datasets is limited. We also found that our model needed only the four most important features to explain 90% of the model output for all instances. This may indicate that the presence of the other features may lead to overfitting. Without the presence of an external dataset, the degree to which these features contribute to overfitting is unknown. Also, only one measurement for IOP at a follow‐up visit was available for each participant. IOP has been shown to have diurnal fluctuations [18] and a single measurement may be inaccurate. IOP trends tend to guide clinical management as opposed to single pressure measurements in an office setting. We also included data from both eyes from a single patient when the data was available and the patient met eligibility criteria. This may have resulted in introducing nonindependence into the dataset as IOP could have been correlated between the two eyes of a patient. Additionally, this study cannot establish a causal relationship between the features included and IOP. SHAP values represent a statistical association between features and model output. SHAP does not measure the strength of the relationship between values of the features and the actual IOP measured for a given patient. Future studies may consider assessing other conventional importance metrics using alternative approaches upon appropriate harmonization. Furthermore, disease coding inaccuracies and the use of self‐reported questionnaires, and the broad definition of taking one medication in the list may introduce potential misclassification bias. The purpose of this study was not to develop a ready‐to‐use model that can be employed in a clinical setting for prognostication but to evaluate the performance of various models based on features that could be accessible in local systems or electronic health records. Also, this study did not include several factors that have an effect on IOP, and thus their effects on model predictions cannot be accounted for. For example, we did not include in our model the length of time which patients were receiving treatment, and we did not control for whether or not patients received surgical management for glaucoma. Information pertaining to these factors was largely missing from the UK Biobank for the cohort of participants with glaucoma who reported taking a glaucoma therapeutic. It is possible that these factors would have played a role in the learning process of the algorithms we compared and may have usurped the importance of other features. Additionally, it is not clear if the IOP measured at follow‐up is indicative of disease progression or diurnal variations.

## 5. Conclusion

By applying explainable machine learning to glaucoma in a large biobank cohort, this study extended prior IOP prediction models on glaucoma surgery to glaucoma medication treatment and indicated the importance of blood LDL and IGF‐1 in contributing to the outcomes of IOP‐lowering therapy in glaucoma patients. We also demonstrated the ability of XGBoost to predict these outcomes. These findings may help guide future research directions to unveil the underpinnings of glaucoma pathogenesis and therapeutic efficacy by identifying factors and interactions that may be involved in glaucoma via prospective and longitudinal studies as well as integrative precision medicine approaches.

**Nomenclature**
IOP: intraocular pressure
LDL: low‐density lipoprotein
IGF‐1: insulin‐like growth factor 1
ML: machine learning
AUC: area under receiver operating characteristic curve
XGBoost: extreme gradient boosting
Light GBM: light gradient boosting machine
DBH: demographic and baseline health
SHAP: SHapley Additive exPlanation

## Conflicts of Interest

The authors declare no conflicts of interest.

## Funding

This work was supported by the National Institutes of Health, 10.13039/100000002, R01‐EY013178, R01‐EY028125, and R01‐EY036070, and Research to Prevent Blindness, 10.13039/100001818.

## Supporting information


**Supporting Information** Additional supporting information can be found online in the Supporting Information section.. Tables S1, S2, S3 and S4: The final hyperparameters and their respective values for each dataset a given model was trained and tested on. Figure S1: A matrix of the interaction and main effect values calculated via SHAP for all features in the combined feature set.

## Data Availability

The data that support the findings of this study are available from UK Biobank. Restrictions apply to the availability of these data, which were used under license for this study. Data are available from the authors with the permission of UK Biobank.
