# Supplementary material for: Predicting Intraocular Pressure From Glaucoma Patients Receiving Medication Treatment Using Explainable Machine Learning
Source: Biomed Res Int. 2026 Jan 30;2026:9930837. doi: 10.1155/bmri/9930837 (PMC12858418; doi:10.1155/bmri/9930837)
Supplement: Supplementary file 1 — Supporting Information Additional supporting information can be found online in the Supporting Information section.. Tables S1, S2, S3 and S4: The final hyperparameters and their respective values for each dataset a given model was trained and tested on. Figure S1: A matrix of the interaction and main effect values calculated via SHAP for all features in the combined feature set. [file BMRI-2026-9930837-s001.docx]

Supplementary Material

**Title: Predicting Intraocular Pressure from Glaucoma Patients Receiving Medication Treatment Using Explainable Machine Learning**

*
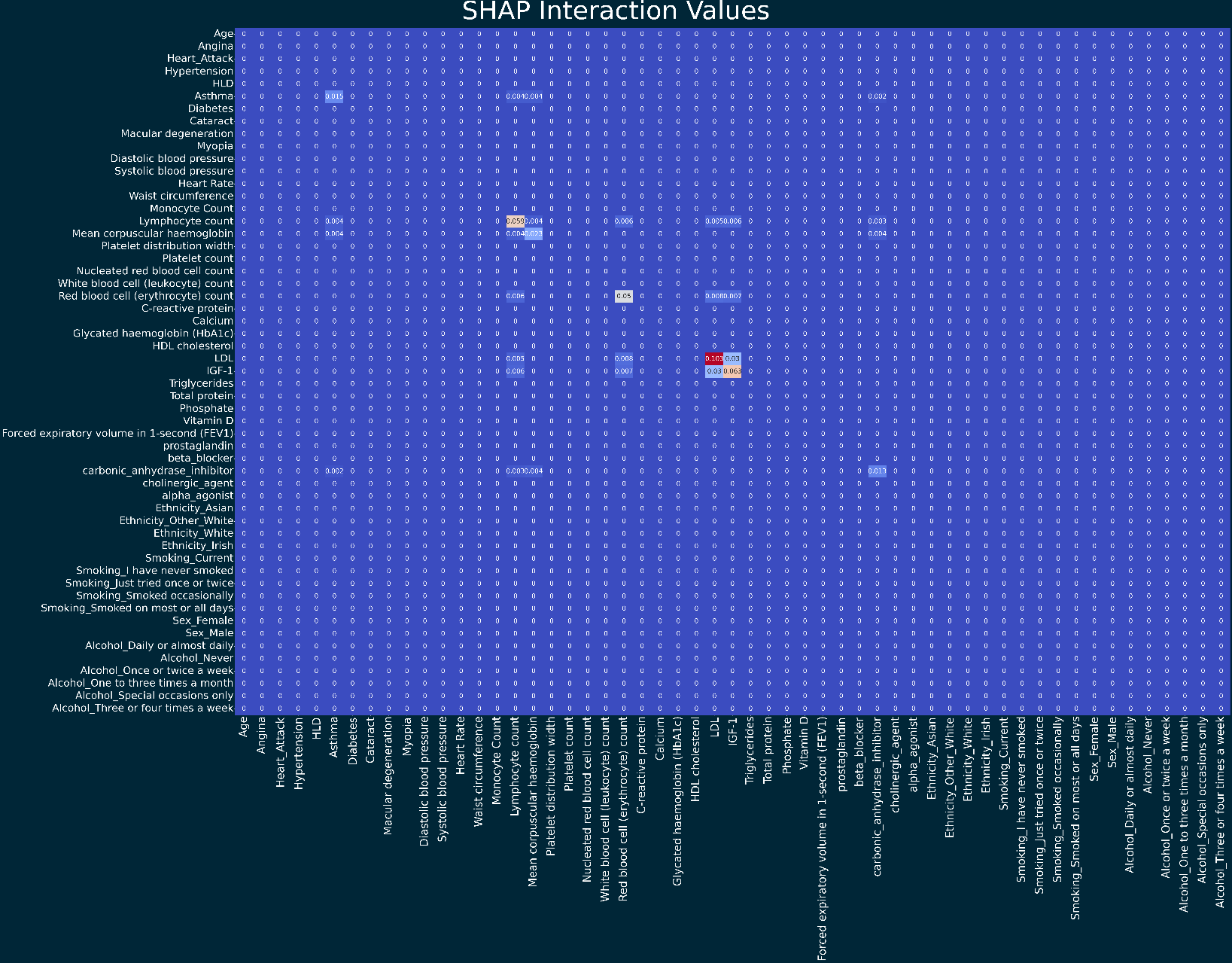
***Authors:** Robert T. James, Wenke Liu, Gadi Wollstein, Joel S. Schuman, David Fenyo, Kevin C. Chan^*^

**Supplementary Figure 1:** A matrix of interaction and main effect values calculated via SHAP for all features in the combined feature set. The diagonal features are main effects values which capture the effect that the value of a given feature alone has on model output after subtracting the additional feature interactions. The off-diagonal values represent the additional effect on model output that occurs between pairwise interactions between features.

**Supplementary Tables: Final Hyperparameter Values.** Hyperparameters and their respective values for each dataset a given model was trained and tested on are depicted in separate tables below. The hyperparameters included the tables are not an exhaustive list of the hyperparameters that could be tuned for each model during training. However, the hyperparameters included are those which were used in the randomized search package to select the final model parameters before measuring performance. For the Logistic Regression model, all parameters used were the in the default settings.

**Supplementary Table 1: Random Forest Hyperparameters**

| Dataset | bootstrap | max_depth | max_features | min_samples_leaf | min_samples_split | n_estimators |
| --- | --- | --- | --- | --- | --- | --- |
| Demographics | True | None | Log2 | 30 | 2 | 70 |
| Physiometabolic | True | None | None | 20 | 20 | 100 |
| Combined | True | None | sqrt | 15 | 5 | 75 |

**Supplementary Table 2: Support Vector Machine Hyperparameters**

|  | C | gamma | kernel | probability |
| --- | --- | --- | --- | --- |
| Demographics | 50 | 1 | sigmoid | True |
| Physiometabolic | 17 | 1.8 | poly | True |
| Combined | 2 | 2 | sigmlid | True |

**Supplementary Table 3: Light Gradient Boosting Machine Hyperparameters**

| Dataset | num_leaves | min_child_samples | min_child_weight | subsample | colsample_bytree | reg_alpha | reg_lambda | max_depth | n_estimators |
| --- | --- | --- | --- | --- | --- | --- | --- | --- | --- |
| Demographics | 15 | 15 | 1 | 0.6 | 0.6 | 7 | 7 | 2 | 200 |
| Physiometabolic | 46 | 26 | 3 | 0.3 | 1 | 1e-1 | 1e-1 | 15 | 200 |
| Combined | 44 | 50 | 1e-3 | 1 | 1 | 3 | 80 | 10 | 175 |

**Supplementary Table 4: XGBoost Hyperparameters**

| Dataset | Booster | Random_state | learning_rate | gamma | subsample | colsample_bytree | reg_alpha | reg_lambda | max_depth | n_estimators |
| --- | --- | --- | --- | --- | --- | --- | --- | --- | --- | --- |
| Demographics | gbtree | 42 | 0.6 | 0.4 | 0.6 | 0.7 | 5 | 7 | 10 | 100 |
| Physiometabolic | gbtree | 42 | 0.6 | 0.5 | 0.9 | 0.5 | 10 | 10 | 5 | 2000 |
| Combined | gbtree | 42 | 0.6 | 0.2 | 0.9 | 0.8 | 10 | 20 | 5 | 20 |
